# Developing medical imaging AI for emerging infectious diseases

**DOI:** 10.1038/s41467-022-34234-4

**Published:** 2022-11-18

**Authors:** Shih-Cheng Huang, Akshay S. Chaudhari, Curtis P. Langlotz, Nigam Shah, Serena Yeung, Matthew P. Lungren

**Affiliations:** 1 Department of Biomedical Data Science, Stanford University, Stanford, CA USA; 2 Center for Artificial Intelligence in Medicine & Imaging, Stanford University, Stanford, CA USA; 3 Department of Radiology, Stanford University, Stanford, CA USA; 4 Department of Computer Science, Stanford University, Stanford, CA USA; 5 Department of Electrical Engineering, Stanford University, Stanford, CA USA; 6 Clinical Excellence Research Center, Stanford University School of Medicine, Stanford, CA USA

**Keywords:** Medical imaging, Infectious diseases, Machine learning

## Abstract

Very few of the COVID-19 ML models were fit for deployment in real-world settings. In this Comment, Huang et al. discuss the main steps required to develop clinically useful models in the context of an emerging infectious disease.

## Teaser

Advances in artificial intelligence (AI) and computer vision hold great promise for assisting medical staff, optimizing healthcare workflow, and improving patient outcomes. The COVID-19 pandemic, which caused unprecedented stress on healthcare systems around the world, presented what seems to be a perfect opportunity for AI to demonstrate its usefulness. However, of the several hundred medical imaging AI models developed for COVID-19, very few were fit for deployment in real-world settings, and some were potentially harmful. This review aims to examine the strengths and weaknesses of prior studies and provide recommendations for different stages of building useful AI models for medical imaging, among them: needfinding, dataset curation, model development and evaluation, and post-deployment considerations. In addition, this review summarizes the lessons learned to inform the scientific community about ways to create useful medical imaging AI in a future pandemic.

The COVID-19 pandemic arose during one of the most innovative periods for biomedical data science, chiefly led by achievements in artificial intelligence (AI), which inspired many efforts globally to leverage digital health data and machine learning techniques to address challenges posed by the pandemic. However, of the innumerable COVID-19-related machine learning efforts developed during the pandemic’s most critical time, almost all failed to materialize demonstrable value, and worse, some were potentially harmful^1–5^. That these failures occurred despite the AI and data science community’s worldwide united attempt to generate, aggregate, share and utilize large volumes of COVID-19-related data, ranging from simple dashboards, to models for populational-wide risk prediction^6,7^, early detection and prognostication^8–12^, severity scoring^13–15^, long-term outcome and mortality predictions^16–19^, is alarming.

**Fig. 1:**
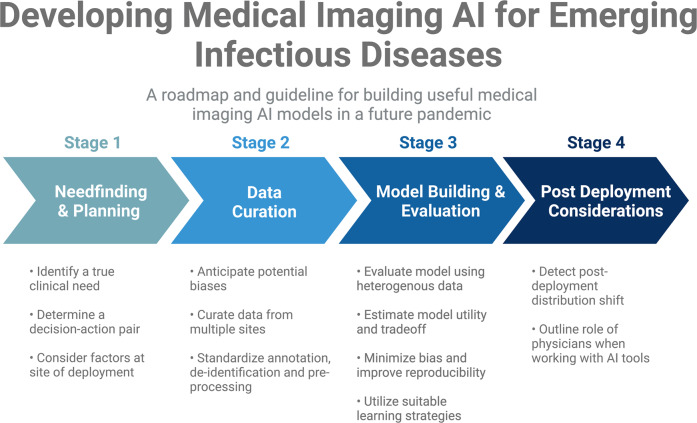
The four stages of building useful medical imaging AI models for emerging infectious diseases. We provide recommendations and guidelines for the four stages of medical imaging AI development process in each section of this manuscript, namely: needfinding, dataset curation, model development and subsequent evaluation, and post-deployment considerations. Each stage of the development process should be considered when building medical imaging AI for emerging infectious diseases.

Given the sustained belief in the promise of AI for medical imaging and the undelivered promises of machine learning to help during the pandemic, it is essential to summarize the lessons that can be learned from this collective experience to prepare for the future. The purpose of this work is to investigate how we could better position ourselves to leverage artificial intelligence for medical imaging to be useful in a future pandemic. Based on current literature, we provide an evidence-based roadmap for how machine learning technologies in medical imaging can be used to battle ongoing and future pandemics. Specifically, we focus in each section on the four most pressing issues, namely: needfinding, dataset curation, model development and subsequent evaluation, and post-deployment considerations (Fig. 1). For each section, we highlight lessons that can be learned from the shortcomings of prior studies and provide recommendations and guidelines to address them.

## Needfinding and planning

“At the root of designing useful tools is the concept of fulfilling a true need^20^”. However, this needs-first principle may sometimes be lost in the process when developing AI models for medicine and healthcare. Likewise, the lack of alignment and understanding of the end user’s needs has contributed significantly to the limited adaptation of COVID-19 AI tools in real-world settings. Instead of consulting clinicians to identify a true need, many COVID-19 models were developed based on the availability of datasets. For instance, many medical imaging AI models were developed specifically for COVID-19 detection using CT and CXR, even though several radiology societies have suggested that imaging tests should not be used independently to diagnose COVID-19^21,22^. Even if these models can achieve high evaluation metrics, their utility is limited in the absence of a genuine clinical need. Therefore, it is crucially important for AI teams to work closely with clinicians and conduct impact analysis on workflows to identify a true need in healthcare settings that can be fulfilled by automation.

Once a clinical need is identified, it is just as essential to determine a specific “action” to pair with the machine learning model’s output to address this clinical need. Most medical image AI models are currently evaluated based on how closely the model’s output matches recorded diagnoses or outcomes^23^, instead of what matters most to healthcare workers and patients - the ability to bring favorable change in care and improve patient outcomes via a specific action. For example, a COVID-19 model capable of identifying patients at high risk of progression to severe COVID-19 can be paired with the action of administering antiviral therapies such as Ritonavir and Remdesevir^24^. Similarly, a model that tracks COVID-19 progression using time-series medical images can be paired with the action of discharging the patient or requiring supplemental oxygen.

By determining a “decision-action” pair^23^, AI developers can instead evaluate the model’s utility based on the estimated net benefit in the context of the clinical need (see Model Building and Evaluation section). In a healthcare system, many factors can influence the best course of action, the net incremental value of taking this action, and whether the action is taken. Therefore, there is a need to clearly define the care pathway for the patient, including the specifics of who is interpreting the model’s output, who is taking action, and how many patients this person can effectively take action for, to determine the optimal decision-action pair. AI developers should continue to consult physicians and healthcare providers to determine the decision-action pair and to estimate the net utility of the action.

It is also crucial for AI developers to thoroughly consider the real-world environment their AI models are intended to deploy at. Many factors at the deployment site, including patient demographics, disease prevalence, clinical workflows, and availability of relevant software or infrastructures, can significantly affect the usability and reliability of the AI model. Take, for example, an AI model that is intended to assist radiologists by highlighting abnormal regions of the medical image. Without infrastructures to integrate this model into the clinicians’ workflow or proper software to overlay the model’s predicted abnormal regions on the original image, the model’s utility is greatly limited. Furthermore, deep learning models are known to be sensitive to distribution shifts, which means that a model’s performance can be significantly impacted if factors at the site of deployment cause input data to deviate from the model’s training data. For instance, models developed without considering patient position and laterality for chest x-rays or slice thickness and protocol for CT scans at the deployment site might result in an unexpected drop in performance and limited utility. Hence, just as important as identifying a genuine clinical need and determining a prediction action pair, carefully considering aspects at the site of deployment can often mitigate unexpected and undesired outcomes.

## Data curation

Curating representative and high-quality data is crucial for building useful machine-learning models. Many medical image datasets were publicly released to encourage other researchers to build COVID-19 AI models. However, a recent systematic review revealed that the majority of more than a hundred datasets the authors identified did not pass their assessments for risk of bias^25^. These biases can cause the machine learning models to learn spurious correlations between predictors and outcomes, and fail to generalize when deployed in hospitals and clinics. For instance, patients with more severe cases of COVID-19 typically get their chest X-rays acquired supine or recumbent in anterior-posterior [AP] projection, whereas healthier patients get imaged while upright (posterior-anterior [PA] projection). Building a model using a dataset that includes images of patients in both AP and PA projections can cause spurious correlations in predicting COVID-19 severity, long-term outcomes, or mortality based on the image projection rather than on semantic image features. These unexpected correlations led to “shortcut learning^26^”, which undermined the model’s performance and might have been anticipated if a medical imaging specialist had been part of the multidisciplinary team designing the model.

One of the most important yet challenging aspects of building medical imaging AI models is curating large-scale datasets with images from various healthcare settings. Without large-scale data from multiple heterogeneous data sources, models can be biased towards specific patient demographics or overfit certain imaging devices’ characteristics, leading to lower performance when deployed at a new institution. However, annotating medical images is time-consuming and cost-prohibitive at scale. Thus, most publicly available COVID-19 medical image datasets that met the criteria for proper risk and bias assessment are relatively small, with only several hundred labeled images^25^. Furthermore, datasets that contain more than a few thousand imaging studies tend to be limited to a particular healthcare system or country^27,28^, likely due to the lack of a standardized strategy to preserve patient privacy when sharing data. To improve model performance and generalizability, many studies splice together data from multiple sources to create “remix datasets”. While this might seem like a promising solution, models that appear promising in the course of development can have lower performance when deployed. For example, the machine learning model might learn to identify the hospital instead of COVID-19 based on pixel distribution of the hospitalʼs imaging hardware or the hospitalʼs watermark and may predict COVID-19 solely based on identifying images from hospitals with high COVID-19 prevalence. Additionally, different datasets might have different standards for annotation (i.e., PCR test results vs. expert opinion) or different tolerance for inter-rater variability, which can increase uncertainty for the model. Therefore, ensuring label integrity and standardization between different sites and datasets is essential.

Data-sharing frameworks that standardize or account for image acquisition, de-identification, pre-processing, annotation, and quality assurance from different hospitals while preserving patient privacy are critical for developing useful models. One notable example is RSNA’s RICORD^29^, a public COVID-19 database with medical images assembled from sites worldwide. This effort clearly defined data inclusion criteria, developed a data de-identification protocol (RSNA anonymizer), and utilized a cloud-based data annotation tool (MD.ai) to standardize data sharing while preserving patient privacy. Furthermore, multiple radiologists were involved in the annotation process to ensure label integrity and ascertain that labels were chosen with clinically-driven goals. Additionally, RICORD requires all sites to provide a detailed description of the data, imaging metadata for each imaging exam, and relevant patient medical data. RICORD is now hosted on the Medical Imaging and Data Resource Center (MIDRC), a linked-data commons explicitly built with AI in mind. Efforts such as RICORD and MIDRC enable easy access to large-scale multi-institutional medical images along with standardized and high-quality annotations, thereby providing more diverse and better representations of patient demographic and imaging acquisition methodology, which may help mitigate potential confounders.

## Model building and evaluation

Thorough and proper evaluation of AI models before deployment is crucial. Without evaluations in different settings and populations, irrational confidence in model’s performance can follow, which can have deleterious consequences when the model is deployed. On top of evaluating models using heterogeneous data from multiple sites and representative data that the models are expected to ingest during deployment, it is also vital to evaluate AI models across different demographic groups. Without representative data or the right training objective, AI models can be biased against specific protected attributes such as age, gender, and race^30^. For example, in CheXclusion, the authors showed that state-of-the-art deep learning models for Chest X-rays are biased to demographic attributes^31^. In addition, Banerjee et al. have shown that medical image AI models can be explicitly trained to predict self-reported races with high accuracy—something even medical experts cannot^32^. This indicates that medical imaging models have the potential to use spurious correlations between patient demographics and the outcome of interest as shortcuts for prediction, which will render the prediction unreliable or even outright harmful. Therefore, methods such as true positive rates, statistical parity, group fairness, and equalized odds should always be considered for detecting algorithmic bias before deployment.

If algorithmic bias is detected for a model, AI developers should collaborate with clinicians to identify the sources of bias and make adjustments to improve the model^33^. Leveraging large-scale data from multiple sources has been demonstrated as an effective strategy for combating the risk of algorithmic bias^31^. However, sharing data across different hospitals while preserving patient privacy might not always be feasible. Alternatively, Obermeyer et al. found that biases are sometimes attributed to label choice since the labels are often measured with errors that reflect structural inequalities. Specifically, the model in their study uses health cost as a proxy for health severity, and since historical data have exhibited reduced healthcare spending on Black patients, the model learned to be biased against Black patients who are as sick as white patients. Instead of modifying the model or the input data, changing the labels used to train the model could address algorithmic bias in certain situations^34^. Other strategies to address model bias^35^ include (1) pre-processing approaches such as oversampling of minority groups^36^, (2) in-processing approaches that add explicit constraints in the loss function to minimize performance difference between subgroups^37,38^, and (3) post-processing approaches, such as equalized odds to correct the outputs based on the individual’s group^39–41^.

In the “Needfinding and planning” section, we emphasized the importance of determining utility via the model’s decision–action pair. Knowing the specific actions associated with a model’s outputs allows us to determine the model’s utility and tradeoff. Unfortunately, most studies chose models based on evaluation metrics such as AUROC and accuracy, which are statistical abstractions that do not directly relate to improvements in medical care^42^. Instead, models should also be evaluated based on the estimated net incremental value or utility of taking specific actions due to the model’s prediction. This allows AI developers to determine directly if a particular action based on a model’s output would bring more benefit or harm to the patient. One way to define utility is by estimating the net dollar value of taking a specific action based on the model’s prediction, including costs of intervention, allocation of resources, and future patient health expenses. Once the utility is defined, models can be evaluated using the indifference curve—a line on the ROC plot that shows combinations of sensitivity and specificity that result in the same utility^23^. Not only should the indifference curve be used to choose the operating point of a model, it should also be used to choose the model to deploy in hospitals and clinics. As stated by Irwin and Irwin, “ROC curves show what is feasible, indifference curves show what is desirable. Together they show what should be chosen^43^”. It is worth noting that a model can have lower AUROC, and yet an operating point with higher utility. Other studies have shown that it is possible to incorporate utility directly into model building and thus achieve tighter alignment to patient outcomes^44^. While a study of net incremental value is not necessary for AI publications, it is crucial to consider the net utility when comparing models for deployment in real-world settings.

Although very few COVID-19 AI models failed to provide clinical value solely due to their learning strategy, it is still important to highlight learning strategies that could significantly benefit model developments for future efforts. In situations where frameworks have been set up to share labeled data across sites, we advocate that data providers also share pertinent medical information to encourage the development of multimodal fusion models. It has been repeatedly shown that patient medical history and laboratory data are typically required to enable physicians to interpret medical images in the appropriate clinical context^45,46^. For instance, many medical characteristics have been linked to COVID-19 severity and long-term outcomes^47^. Thus, building machine learning models without these patient medical records could limit the capabilities of an AI model. In fact, a systematic review has shown that medical image models that fuse data from multiple modalities generally lead to increased performance compared to the performance of single modality models^48–51^. Similarly, several COVID-19 medical imaging models incorporating clinical variables have observed improvement in performances over imaging-only models^8,11^.

If the appropriate data-sharing framework is unavailable, models can still be trained across multiple institutions without explicitly sharing data using federated learning - a collaborative machine learning paradigm. Instead of bringing data to the machine learning model, the model is distributed and trained within the firewalls of each institution^52^. Existing studies have already demonstrated that a federated learning framework can lead to nearly the same performance as that of models that use centralized data or ensembling strategies^53,54^. More recently, blockchain-based frameworks allowed decentralized learning without a central coordinator, promoting equality in training multicentric models^55^. Several software frameworks for enabling federated learning in real-world settings already exist, including NVIDIA CLARA^56^, Intel OpenFL^57^, and Flower^58^. Furthermore, federated evaluation platforms, such as MLPerf^59^, allow developers to evaluate the generalizability of their models across multiple sites while preserving patient privacy.

While an abundance of medical images exists in most healthcare institutions, training machine learning models using the traditional supervised paradigm requires annotations by medical experts and thus is cost-prohibitive at scale. Self-supervised pre-training strategies allow machine learning models to obtain supervisory signals from the data without explicit labels, thus allowing models to learn from all available data even if some are not annotated^60^. Furthermore, after the self-supervised pre-training stage, these models can be fine-tuned for many downstream tasks with limited number of labels. Studies have shown that medical image models pre-trained using self-supervised learning are robust to distribution shift and require fewer labels during supervised fine-tuning^61,62^. Recent studies have also demonstrated the possibility of multimodal self-supervised learning for medical images by leveraging the corresponding radiology reports, and have demonstrated superior performance using only 1% of the training labels compared to supervised models^63,64^. Yan et al. have further proposed training self-supervised models with data from multiple healthcare centers using Federated Learning and have shown improvement in robustness and performance over models trained using data from a single institution^65^.

Explainable artificial intelligence (AI) techniques, including saliency maps, generative adversarial networks (GANs), and counterfactual explanations (CE), can be used to identify spurious correlations and confounders the model relies on to make predictions. For example, by using saliency maps and GANs, DeGrave et al. found that models rely on regions outside of the lung fields, such as laterality markers, to make predictions on COVID-19 datasets. While prior work has argued that saliency should not be used for explanation^66^, counterfactual explanation techniques have been shown to reveal learned correlations to the model’s prediction. By applying minimal but meaningful perturbations of an input image to change the original prediction of a model, CEs can provide explanations that are understandable by humans. In addition to detecting spurious correlations, explainable AI techniques can also reveal human-subliminal signals about the disease and give us a better chance of addressing challenges posed by the pandemic. Lastly, slice discovery methods^67,68^, such as Domino^69^, can identify and describe semantically meaningful subsets of data on which the model performs poorly, thereby revealing spurious correlations.

## Post deployment considerations

Even after a model is thoroughly evaluated and deemed suitable for deployment, several challenges can still impact the model’s ability to bring favorable change for patients post-deployment. One such challenge is post-deployment distribution shift. Most medical image models are trained on a static dataset curated from a specific label definition, patient population, timeframe, scanner type, and protocol. However, real-time changes in hospital workflow or disease prevalence may alter the model’s input data distribution, causing models’ performance to degrade. There are two major types of distribution shift: concept shift and data shift^21^. Concept shift includes the arrival of a novel class or class evolution, such as bacterial pneumonia vs COVID-19 pneumonia. Data shift can have many causes, including new imaging modalities, new versions of software, or adjustments to data acquisition procedures. For example, if a COVID-19 model is trained to predict patient mortality with pre-vaccination COVID-19 patient data, it would dangerously overestimate the risk of death in real-world data during deployment after the vaccine was widely distributed. One way to detect distribution shifts is by monitoring the model’s performance longitudinally and checking for statistically significant performance drops. While this method effectively detects label shifts, it is challenging to implement in practice as it requires periodic data annotation. A more tangible approach is based on detecting shifts in the input data distribution. This can be done by measuring differences between real-world and training data using two-sampled-test-based statistical approaches^70^. Alternatively, methods for modeling predictive uncertainty, such as Prior Networks^71^, can be used to detect data shifts in real time. Hospitals should establish protocols and procedures for adjusting the model as soon as a distribution shift is detected to maintain optimal model performance and patient safety.

Another challenge is clearly outlining the role of physicians when working with AI tools. Some existing literature has argued that the role of radiologists in the AI era is to become educated consumers of AI tools by identifying clinical needs for AI, evaluating AI tools before deployment, and maintaining their expertise by avoiding overreliance on technology^72–74^. Furthermore, it is crucial to educate clinicians about the limitations of AI. This allows clinicians to help identify potential distribution shifts, either by reporting conflicting diagnoses between that made by the model and that made by themselves or by noting changes in their medical practice^75^. Hospitals should also make sure concerns about an AI tool’s functionality or factors that can potentially change data or label distributions can be easily reported by clinicians. In addition, educating radiologists on the limitations of AI can prevent them from blind acceptance of the AIʼs output. Over-reliance on AI tools can diminish the physician’s perspective and diagnostic skills. Furthermore, a machine learning tool is typically limited to the few diagnoses it is intended for. It is, therefore, still important for radiologists to look for other abnormalities in the image that the model cannot detect. In other words, clinicians should still make their independent diagnoses and use AI tools only as supplements or aids.

## Conclusions

While advancements in AI for medical imaging hold great promise in improving healthcare, many overlooked aspects of the model development process and use lifecycle have hindered large-scale deployment during the most critical time of the COVID-19 pandemic. Independent of the choice of learning strategies or model architectures, many COVID-19 AI models were unfitting for use due to shortcomings in needfinding, data curation, model evaluation, and post-deployment considerations. We urge researchers to be mindful of the aforementioned potential biases and limitations currently hindering the deployment of medical imaging AI models. In addition, considerable planning is necessary to prepare for future health crises, including developing data-sharing frameworks and implementing AI deployment infrastructures in hospitals. We hope this review will inform the data science and healthcare community about strategies for building useful medical imaging AI models for future infectious diseases.
